# From Probiotics to Postbiotics—An Update on Their Biotherapeutic Potential and the Emerging Strategies in Human Health

**DOI:** 10.3390/ijms27052218

**Published:** 2026-02-26

**Authors:** Nicoleta Maricica Maftei, Lenuța Ambrose, Elena Dogaru, Răducu Răileanu, Oana Laura Mierlan, Octavian Amariței, Ana Ramos-Villarroel, Gabriela Isabela Răuță Verga, Tudor Vladimir Gurău, Gabriela Gurău

**Affiliations:** 1 Research Centre in the Medical-Pharmaceutical Field, Faculty of Medicine and Pharmacy, Dunarea de Jos University of Galati, 800008 Galati, Romania; naron@ugal.ro (N.M.M.); lenuta.ambrose@ugal.ro (L.A.); dr.elena.pogorevici@gmail.com (E.D.); laura.mierlan@ugal.ro (O.L.M.); octavian.amaritei@ugal.ro (O.A.); gabriela.verga@ugal.ro (G.I.R.V.); gabriela.gurau@ugal.ro (G.G.); 2 Sf. Ioan Emergency Clinical Hospital for Children, 800487 Galati, Romania; 3 Sf. Apostol Andrei County Emergency Clinical Hospital, 800578 Galati, Romania; 4 Venezuelan Institute for Scientific Research (IVIC), Caracas 1204, Venezuela; ay2170@gmail.com; 5 Monagas Campus, University of Oriente, Maturín 6201, Venezuela

**Keywords:** probiotics, postbiotics, human health, biotherapeutic, gut microbiota

## Abstract

Probiotics and postbiotics have gained increasing attention in microbiome research due to their potential roles in maintaining gut homeostasis and supporting human health. While probiotics are defined as live microorganisms that confer health benefits when administered in adequate amounts, postbiotics represent preparations of inanimate microorganisms and/or their components that also exert biological effects on the host. This narrative review provides an updated overview of the current knowledge on probiotics and postbiotics, with a particular focus on their mechanisms of action, production strategies, and emerging applications in human health. The review discusses key mechanisms through which probiotics and postbiotics interact with the host, including modulation of the gut microbiota, enhancement of epithelial barrier integrity, immune system regulation, metabolic modulation, and systemic signaling pathways. Advances in production technologies, ranging from conventional fermentation to innovative inactivation and stabilization approaches, are also examined, alongside challenges related to yield optimization, stability, safety, and standardization. Although a growing body of evidence suggests potential benefits of probiotics and postbiotics in metabolic, inflammatory, gastrointestinal, and immune-related conditions, much of the available data is derived from preclinical studies or small-scale clinical trials. Consequently, their clinical efficacy, optimal dosing, and long-term safety require further validation. By integrating current findings and highlighting existing gaps in the literature, this review aims to clarify the therapeutic potential of probiotics and postbiotics and to support the development of more robust, evidence-based strategies for their application in functional foods, supplements, and future biotherapeutic interventions.

## 1. Introduction

At the start of the new century, the health of intestinal flora has emerged as a major concern among researchers. The number of intestinal flora is vast and more complex in comparison with the symbiotic flora in other parts of the human body. Approximately 3.9 × 10^13^ is the total number of intestinal flora, for adults, in the colon, somewhat more than the total number of human cells [1]. The host is aided in digesting nutrients in food by the intestinal flora which also participates in systemically physiological activities of the human body, which are in close relation with human health [1]. Disruption of gut microbiota homeostasis, known as dysbiosis, has been associated with the production of many metabolic diseases such as inflammatory bowel disease (IBD), obesity, type 2 diabetes, cardiovascular disease, certain cancers, and neurodegenerative disorders. This highlights the need for effective methods to foster and support this delicate microbial ecosystem, and functional dietary components, termed probiotics and postbiotics, have attracted increasing attention in this context [2,3,4,5]. On the other hand, the conserved functional characteristics of microbial species vary significantly between individuals. Also, health status or host benefits do not necessarily reflect microbiome diversity. On the contrary, optimal performance is imposed by age and sex [6], and a healthy microbiota functions as a barrier within the microbial community, defending the body from external and internal aggressions (antibiotics, infections and stress), promptly returning from dysbiosis (a disturbed state) to eubiosis (a homeostatic state) [7].

Finally, early programming of the microbiota and immune system during pregnancy, childbirth, lactation and weaning is also very important, determining adult immune function, the microbiome and health [8]. On the other hand, worldwide, a rapid increase in the number of chemical and biological substances that advertise their ability to affect the functions and composition of microbiota for the benefit of human health has been observed [9]. An evolving concept—named the personalized microbiome”—is that of improving human health by modulating microbial interactions at all stages of life. This concept is increasingly significant for consumers, food manufacturers, as well as health professionals and regulatory authorities. Regarding dietary interventions to modulate the microbiota, these include numerous fermented foods or functional foods but also high-fiber diets (prebiotics), as well as probiotics and symbiotics, and some of them are available in the form of foods, drugs and medical devices [10,11,12,13,14]. Regarding drugs, synthetic drugs have evolved into the central element of the 21st centuries therapeutic strategies, offering novel therapeutic approaches for a multitude of conditions [15]. Capitalizing on methods used in molecular biology, chemistry, pharmacogenetics and pharmacogenomics, synthetic drugs target new pathways with remarkable specificity, presenting a high degree of efficacy and predictability in treating several conditions [16]. Due to their capacity for personalized design, synthetic drugs have reshaped, are reshaping and will reshape the entire therapeutic landscape, as they can provide solutions where traditional treatments have had limited impact [17]. Although synthetic drugs can address specific symptoms, they have the disadvantage that they may not properly restore microbial balance, thus providing only symptomatic relief without addressing the underlying cause [18]. In addition, synthetic drugs, especially broad-spectrum antibiotics that attack nonspecifically, can exacerbate health problems associated with gut dysbiosis [19]. While synthetic drugs constitute a great step forward in the treatment of disease, their interaction with the gut microbiota is a key point that presents significant challenges that require a more holistic approach [20]. Given the ability of probiotics and postbiotics to modulate the gut microbiota, maintain health, and treat diseases, it is very important to understand their effects and mechanisms. A frontier in biomedical research is the advancement of their clinical application. Although probiotics and postbiotics are classified as health products rather than drugs, public acceptance and regulatory norms restrict their administration in the medical field.

This review was conducted as a narrative, non-systematic review of the literature, and aimed to summarize and contextualize current knowledge on the roles of probiotics and postbiotics in human gut health and gut barrier integrity. Although various studies have mentioned that human gut microbiota plays a vital role in health, current research emphasizes the importance of their combined effects, which are less evaluated. For this reason, this review updates current understanding by integrating conclusions on the mechanisms and clinical relevance of the two types of biotics, addressing gaps in the previous literature and underlining their potential utilizations in disease prevention and gut barrier support.

## 2. Terminology and Classification

### 2.1. Probiotics

Probiotics are defined, according to the International Scientific Association for Probiotics and Prebiotics (ISAPP), as live microorganisms that confer a health benefit when administered in adequate amounts. They can support health in various ways: helping the immune system function properly and aiding digestion or increasing nutrient absorption, and some probiotics can help reduce certain digestive symptoms (example: diarrhea associated with antibiotics). A microorganism must be alive when administered to be considered a probiotic and must be documented to have a health benefit. It must also be administered in doses high enough to confer the benefit for which it was produced [21]. Currently, the most used probiotic strains integrate various species of *Lactobacillus*, *Bifidobacterium* and *Saccharomyces* [22]. These species can be acquired in pure form from various pharmaceutical companies or as key components of everyday foods, especially fermented products (e.g., cheese, yogurt and beer, etc.) [23]. Lactic acid bacteria (LAB) and bifidobacteria are Gram-positive anaerobic microorganisms that produce lactic acid and other antibacterial compounds. They colonize the digestive tract and carry out lactic acid fermentation, which is the main component of probiotics. These valuable bacteria colonize the intestine, outcompete pathogenic microbes in competition for nutrients and adhesion sites and, through competitive exclusion, inhibit the growth of pathogens. Probiotics can also secrete antimicrobial substances (bacteriocins and hydrogen peroxide), which can suppress harmful bacteria [24], modulate the host immune response by increasing the production of anti-inflammatory cytokines and promoting the activity of regulatory T cells (helping to maintain immune homeostasis) [24,25]. It is important to emphasize that compared to probiotics, the intestinal microbiota is composed of different kingdoms of microorganisms belonging to different phyla. The most important phyla present in the intestine include the following species: *Actinobacteria*, *Firmicutes*, *Bacteroidetes,* *Actinobacteria*, *Verrucomicrobia*, *Proteobacteria* and *Fusobacteria*. Of these, 90% of the intestinal microbiota is composed of *Firmicutes* and *Bacteroidetes*, and genera such as *Bacillus*, *Enterococcus*, *Clostridium*, *Ruminicoccus* and *Lactobacillus* make up the *Firmicutes* phylum, while the genera *Bacteroides* and *Prevotella* are the predominant genera present in *Bacteroidetes*. The *Actinobacteria* phylum is largely represented by the genus *Bifidobacterium* [26].

Traditional probiotic bacteria (*Lactobacillus* spp. and *Bifidobacterium* spp.) are generally recognized as safe and are administrated in many diseases, but they are not disease specific. For this reason, a crucial new research trend has the aim of characterizing more efficacious and specific next-generation probiotics (NGPs), such as *Akkermansia muciniphila*, *Bacteroides fragilis*, *Bacteroides thetaiotaomicron*, *Prevotella copri*, *Roseburia intestinalis*, *Christensenella minuta*, *Eubacterium hallii*, *Clostridium butyricum*, *Parabacteroides goldsteinii*, and *Faecalibacterium prausnitzii* [27].

### 2.2. Postbiotics

According to ISAPP, microorganisms do not have to be alive to have health benefits. The definition of postbiotics states that it is a preparation of inanimate microorganisms and/or their components, which must confer a health benefit to the host. In short, postbiotics are non-living microbes, whole or in part, that can be consumed for their health benefits to the body [21]. In turn, different researchers have proposed different terminologies to describe postbiotics: paraprobiotics (non-living probiotics), inactivated probiotics, ghost probiotics and metabiotics [28,29].

Postbiotics may include deliberately inactivated, non-viable, and intact microbial cells, meaning microorganisms that are molecularly characterized and accompanied by a detailed description of the inactivation procedure, as well as microbial cell components. These components can consist of cell fragments and cell wall compounds, including peptidoglycans and teichoic acids, structures and substructures such as plasma membrane lipids, surface structures such as flagella, pili, and fimbriae, and extracellular biopolymers or exopolysaccharides. They may also include, with or without associated microbial metabolites or end products, short-chain fatty acids such as acetate, butyrate, and propionate, tryptophan metabolites, organic acids such as indole-3-lactic acid, bacteriocins, vitamins, and antioxidative enzymes [30,31,32,33,34].

Postbiotics have been classified according to various factors, such as

(a) by molecular weight—high molecular weight and low molecular weight postbiotics
(b) by structure—peptides, teichoic acids and plasmalogens [35]
(c) by composition—postbiotics may include carbohydrates such as teichoic acids; lipids such as dimethyl acetyl-derived plasmalogens, acetate, butyrate, propionate, and lactate; proteins such as lactocepin and the p40 and p75 molecules; vitamins such as B-group vitamins; and other complex molecules, including peptidoglycan-derived muropeptides and lipoteichoic acids [36]
(d) by their physiological function—postbiotics with anti-obesogenic, anti-inflammatory, hypocholesterolemia, antihypertensive, antioxidant and antiproliferative effects and exhibiting immunomodulatory properties [36].

It is important to emphasize that the effectiveness of probiotics depends on the administration of specific doses, for example, containing 10^6^–10^7^ colony-forming units per gram of viable cells, but for postbiotics, a specific dose as specified in probiotics has not been identified [37]. Finally, postbiotics are obtained by inactivating probiotic microorganisms. It is important to emphasize that the inactivation method applied to produce postbiotics must be able to preserve the beneficial effects offered by the living form. In this regard, the use of different inactivation methods has been indicated, such as sonication, extraction with solvents and chemicals (e.g., formalin), and enzymatic processes. These methods can be used both at the laboratory and industrial level [38]. Taking these things into account, the most used methods for inactivating probiotic microorganisms are thermal treatments and the temperature and duration of thermal inactivation differ depending on the characteristics of microorganisms (e.g., vegetative cells or spores, growth stage, growth medium, water activity, etc.) [39].

## 3. Mechanism of Action

### 3.1. Probiotics

**Figure 1:**
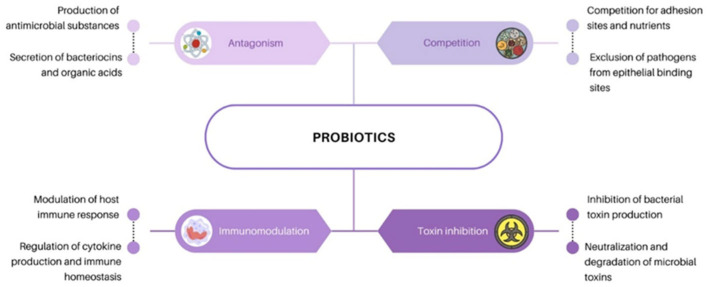
Mechanism of action of probiotics.

In recent decades, considerable progress has been made in the domain of probiotic studies, with an emphasis on the selection and characteristics of individual probiotic cultures, their possible use, and their effect on health. The mechanisms of action of probiotics are related to their influence on the microbiota of the gastrointestinal tract (GI). Approximately 100 trillion microorganisms live in the GI tract—bacteria, viruses, fungi, protozoa—from at least 1000 different species [40], mainly lactobacilli and streptococci. These are found in the stomach and duodenum, where gastric acid, bile, and pancreatic juices inhibit or eradicate most microorganisms. In the intestine, the number of microbes increases progressively, from 10^4^ cells/g in the jejunum to 10^6^ cells/g of contents in the distal ileum, the colon being the most populated region of the GI tract containing up to 10^12^ cells/g of intestinal contents [41]. The diversity of the microbiota between individuals is striking, with every individual having their own distinctive pattern of microbial composition. The composition of the microbiota is determined by genotype, initial colonization at birth, and dietary habits. In the human gut, two bacterial divisions predominate: *Bacteroidetes* and *Firmicutes*, yet the populations of colonizing microorganisms differ between healthy individuals and those with disease or poor health [40]. The microbial composition also appears to differ by age, race, sex, and geographic region [40]. Probiotics have multiple functions in human organisms, their main benefit being the effect on the evolution of the microbiota in the body, ensuring an appropriate balance between pathogens and bacteria required for normal functioning of the body [42,43]. Live probiotic microorganisms are widely used in the production of functional foods and, in some cases, for food preservation, leveraging their ability to inhibit undesirable microbial growth. One of their clinically relevant functions is the restoration of intestinal microbiota following antibiotic therapy, helping to reduce the incidence and severity of antibiotic-associated diarrhea (AAD) [44,45]. Additionally, probiotics contribute to maintaining intestinal homeostasis by limiting colonization of pathogenic microorganisms through competitive exclusion, production of antimicrobial compounds, and modulation of the host immune response [44,45]. Consequently, probiotics can effectively inhibit the development of pathogenic bacteria, such as *Escherichia coli* [46], *Salmonella enteritidis* [47], *Clostridium perfringens* [48], *Campylobacter jejuni* [49], *Staphylococcus* [50], various species of *Shigella* [51] and *Yersinia* [52], thus preventing food poisoning. It has also been confirmed to have a positive effect on digestive processes, the treatment of food allergies [53,54], candidiasis [55] and dental caries [56], increasing the efficiency of the immune system, improving the absorption of vitamins and mineral compounds and can stimulate the generation of organic acids and amino acids [57,58]. Probiotics such as *Lactiplantibacillus plantarum* [59], *Limosilactobacillus reuteri* [60], *Bifidobacterium adolescentis* and *Bifidobacterium pseudocatenulatum* [61] are natural producers of vitamins of group B (B1, B2, B3, B6, B8, B9, B12) [62]. Probiotics are also capable of producing enzymes, such as esterase, lipase, and coenzymes A, Q, Nicotinamide Adenine Dinucleotide (NAD), and Nicotinamide Adenine Dinucleotide Phosphate (NADP). Some products of probiotic metabolism may also exhibit antibiotic (acidophilin, bacitracin, lactacin), immunosuppressive, and anticancer properties [58,63,64,65]. The determination of the basic elements of the beneficial effect of probiotics has been possible through molecular and genetic studies. Four mechanisms have been proposed on the basis of these studies: antagonism through the production of antimicrobial substances [66]; competition with pathogens for adhesion to the epithelium and for nutrients [67]; inhibition of bacterial toxin production [68]; and host immunomodulation [69] (see Figure 1).

Through the four mechanisms, probiotics could have additional broad impacts on human health and disease, but it seems improbable that each probiotic will have properties of all four mechanisms concurrently and constitute a universal remedy for diverse diseases. For this reason, a major role in the action of probiotics are the species- and strain-specific features: cell structure, cell surface, size, metabolic properties and secreted substances. An enhanced protection offered by a biotherapeutic product is the result of a combination of probiotics that demonstrate different mechanisms of action [70].

### 3.2. Postbiotics

**Figure 2:**
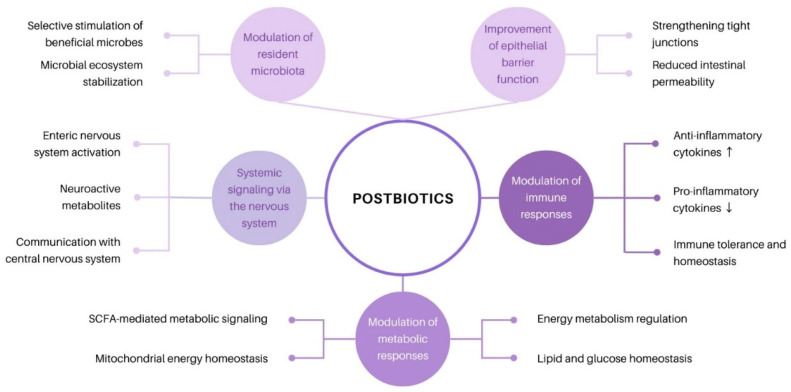
Mechanism of action of postbiotics.

To date, research on the physiological functions of postbiotics has focused specifically on macro-level analysis and evaluation, so the mechanisms of action have not been precisely and fully clarified [71]. Because postbiotics can be a mixture of multiple active substances, they can be driven and mediated by many different mechanisms in terms of effects on the health of the target host. To define the scope of postbiotics and identify potential mechanisms of action, ISAPP convened an expert panel that suggested five main mechanisms of action: modulation of local and systemic immune responses; modulation of metabolic responses; modulation of the resident microbiota; improvement of epithelial barrier functions; and systemic signaling via the nervous system (Figure 2) [71].

Current evidence indicates that postbiotics may exert beneficial effects on cardiometabolic parameters, including reductions in blood pressure, serum cholesterol levels, and body weight. Additionally, postbiotics demonstrate antimicrobial activity mediated by bioactive molecules capable of modulating the composition and function of the resident microbiota. These molecules can also modulate bacteria indirectly through quorum sensing, cross-feeding, and adhesion [71]. Modulation of systemic immune responses by molecules such as histamine, keto acids, short-chain fatty acids (SCFAs), and indole derivatives enables interactions with microbial-associated molecular patterns [9]. Postbiotics can also exert their effects through different mechanisms, involving peptides, SCFAs, vitamins, teichoic acids, enzymes and plasmalogens [72]. Thus, peptides inhibit cell wall synthesis and have antimicrobial properties affecting the brain and intestines, and SCFAs serve as an energy source for epithelial cells (affecting the colon and liver). Vitamins are crucial compounds for colonic thiamine absorption and help release riboflavin, affecting the heart and colon. Teichoic acids also play a vital role in antibiotic resistance and antitumor activity, thus affecting bone marrow and dendritic cells [72]. Enzymes are biochemical compounds that can decompose polymeric substances and eradicate biofilms, thus reducing reactive oxygen species (ROS) and affecting the intestines, pancreas and skin. Studies have shown that postbiotics can also indirectly contribute to wound healing within the intestine [71]. Thus, Isozaki et al. [73] showed in a study that *Levilactobacillus brevis* improved the integrity of the epithelial barrier by enhancing platelet accumulation at the wound site [73]. Other studies have demonstrated that through vitamins and SCFAs derived from microbes (which may act as metabolic modulators), postbiotics positively affect host metabolism and signaling pathways. For example, propionate can increase insulin sensitivity and glucose tolerance but can also alter lipid metabolism [74]. Although the precise mechanisms of action have not yet been established, postbiotics appeared to positively influence respiratory diseases, alcohol-induced liver disease, skin diseases, intestinal diseases, and dental caries [75]. Postbiotics have both local and systemic mechanisms of action. These mechanisms support the survival of epithelial cells, play a central role in facilitating communication between certain bacteria and the immune system, and contribute to maintaining balance and promoting beneficial health [36]. Given the increasing appeal in postbiotic research in recent years, it is possible that various applications for human health and the mechanisms of action underlying them will be discovered.

## 4. Probiotics and Postbiotics in Human Health

### 4.1. Benefits of Probiotics

#### 4.1.1. Probiotics-Clinical Practice

Probiotics play a crucial role in maintaining intestinal health and managing diseases by modulating the intestinal microbiota, improving intestinal barrier functions, and enhancing immune responses. The therapeutic use of probiotics shows promise in the prevention of necrotizing enterocolitis (NEC) by modulating the gut microbiota. Probiotics, (especially *Lactobacillus* and *Bifidobacteria*), can improve epithelial barrier function by upregulating tight junction proteins (strengthening the gut barrier), can support the growth of beneficial commensal bacteria, and can attenuate inflammation by inhibiting the nuclear factor kappa, an enhancer of the light chain of the activated B cells (NF-κB) pathway [75,76]. These findings supporting the beneficial effect of probiotics have been observed in meta-analyses of randomized and observational studies using real-world data (RWD) [77].

Many studies have shown that probiotics may have cholesterol-lowering effects when administered in sufficient amounts [78,79]. For example, Cardoso Umbelino Cavallini et al. [80] administered *Enterococcus faecium* CRL 183 and *Lactobacillus helveticus* 416 to 49 men with hypercholesterolemia to evaluate the effect of both probiotics on lowering lipid profiles [80]. In the study, three groups of participants received different treatments: one group of participants received nothing but a probiotic soy product (SP group); the second group received a soy product containing probiotics with isoflavones (ISP group), and the placebo group received unfermented soy products (USP). The results showed a significant reduction in total cholesterol (by 13.8%), high-density lipoprotein cholesterol (HDL-C) (by 14.7%), and low-density lipoprotein cholesterol (LDL) (by 24%) in the ISP group. However, HDL-C levels remained the same, and the groups receiving either SP or USP treatment did not show a decrease in serum lipid profiles during treatment [80]. The effect of probiotics in improving hyperlipidemia in this study was attributed to several actions: bile salt deconjugation, production of SCFAs due to fermentation of indigestible carbohydrates, and modulation of microbiota, including cholesterol assimilation [81]. In another study, conducted by Yoon et al. [82], researchers investigated the effect of *L. johnsonii* BFE6154 on cholesterol regulation and its mode of action. Researchers observed that stimulation of intestinal epithelial Caco-2 cells with *L. johnsonii* BFE6154 negatively regulated the expression of the Niemann-Pick C1-like 1 (NPC1L1) gene due to activation of the hepatic X receptor (LXR). The researchers also observed an increase in LDL receptor gene expression due to stimulation of HepG2 cells by metabolites produced by *L. johnsonii* BFE6154. Yoon et al. [82] concluded that the results obtained from the experiments suggest that the *L. johnsonii* BFE6154 strain, by regulating cholesterol metabolism in the intestine and liver, could prevent diet-induced hypercholesterolemia. On the contrary, in the study, conducted by Peters et al. [83], the researchers stated that during treatment with the *L. rhamnosus* BFE5264 strain, cholesterol efflux was significantly increased [83]. The increase was attributed to the upregulation of ABCG5/8 (the main neutral sterol transporter in hepatobiliary and transintestinal cholesterol excretion) by activation of liver X receptors (nuclear receptors that are activated by endogenous oxysterols, oxidized derivatives of cholesterol) [84].

Probiotics have also shown significant potential in the management of obesity and associated metabolic disorders through various mechanisms. SCFAs stimulate the release of gut hormones, such as glucagon-like peptide-1 (GLP-1) and peptide YY (PYY), which increase satiety and reduce food intake [85]. A 12-week randomized, double-blind, controlled trial [86] reported that the consumption of a supplement based on *L. plantarum* K50 (LPK) (a strain isolated from kimchi) had numerous positive effects on healthy individuals with a body mass index (BMI) of 25 to 30 kg/m^2^ [86]. The positive effects observed included reductions in triglyceride and cholesterol levels and changes in the gut microbiota, and was characterized by an increase in *L. plantarum* and a decrease in *Actinobacteria*, and changes in obesity [86]. The reductions in lipid profiles caused by LPK were justified by increases in SCFA and changes in the composition of the gut microbiota [86].

In other studies, probiotics have been proposed as a therapeutic supplement for patients with type 2 diabetes to decrease dyslipidemia and promote better metabolic control. Through their immunoregulatory properties, probiotics form the potential mechanism of their action in the control of type 2 diabetes [87,88,89,90]. However, certain studies have not reported substantial changes in lipid profiles following probiotic consumption [91]. The existence of these conflicting results imposes an urgent need for more long-term human studies. Although studies on probiotic strains that improve lipid profiles and metabolic indices are promising, further validation is needed to confirm consistent and long-term therapeutic efficacy.

From our point of view, the inconsistency arises from certain determinants such as population and baseline characteristics, methodological heterogeneity, strain-specific biological activity and dietary and lifestyle factors. The literature does not yet offer a single consensus framework that fully eliminates disparities; as such, we emphasize the need for well-powered, longer duration RCTs with standardized protocols and dietary control, alongside strain-specific sub analysis to resolve the discrepancy.

Finally, a summary of the key findings on various probiotic species and their implications on human health is presented in Table 1.

Guideline-based probiotic dosing is strain- and population-specific. ESPGHAN recommends ≥10^10^ CFU/day of *L. rhamnosus* GG or 5–10 × 10^9^ CFU/day of *S. boulardii* for pediatric antibiotic-associated diarrhea [116], while WGO guidelines indicate typical effective ranges of 10^9^–10^10^ CFU/day for LGG and *B. lactis* BB-12 and 10^8^ CFU/day of *L. reuteri* DSM 17938 in infants [41]. In elderly individuals, clinical studies support higher multistrain doses of 10^10^–10^11^ CFU/day [117].

#### 4.1.2. Probiotics in Food

On the market, probiotic products are mainly available in the form of dietary supplements (including tablets, capsules, powders, liquids and other formulations), added to foods and beverages obtained by fermentation. According to Medical Probiotics Market Size [118], the medical probiotics market was valued at USD 39.32 billion in 2022 and is expected to reach USD 64.13 billion by 2031, at a compound annual growth rate (CAGR) of 5.76% during the forecast period of 2023–2031. Due to increasing health awareness among consumers, increased knowledge about the benefits of probiotics, an aging population, and the prevalence of digestive problems, the probiotics market is continuing to grow. The progress of the probiotics industry can be attributed to increasing health concerns, the expansion of the number of vegans, the increase in demand for plant-based products, and the increase in the number of probiotic supplements targeting inflammatory skin conditions such as eczema and atopic dermatitis [118]. Regarding the form of consumption of probiotics, they can be found naturally in fermented foods (yogurt, kefir, kimchi, boza, etc.) or can be consumed in the form of supplements. Fermentation processes in the food industry have been widely studied and have been reported to provide a wide range of foods with health and nutritional benefits. Fermented foods (food or beverages) are usually defined as products obtained from a slow, controlled microbial cultivation process, and their enzymatic activities on raw substrates are derived from plant or animal sources [119]. Foods prepared in this way use substrates from a variety of agricultural sources containing fiber from cereals/legumes/beans/fruits as a source of prebiotics and some strains of LAB present as fermenting microorganisms. Fermented roots, tubers and leafy vegetables have improved digestibility compared to their consumption in raw form [119]. Microorganisms that promote the fermentation process can secrete postbiotics that suppress the growth of unfavorable bacteria that could otherwise cause food spoilage and disease. An example of this is *Lactobacillus* species, which produce lactic acid, which facilitates the digestion of lactose in milk. Fermentation produces antimicrobial substances: acids, carbon dioxide and alcohol, and the probiotic fermentation byproducts play a role in maintaining a balanced ecosystem while supporting digestion. Fermentation is also one of the oldest methods of food preservation and an excellent way to populate the gut with healthy bacteria [120]. Most probiotic foods contain lactobacilli and/or bifidobacteria, with enterococci being rarely used. The microorganisms used as probiotics are mainly bacterial strains of members of the Lactobacilli group (*L. acidophilus*, *L. plantarum*, *L. reuteri*, *L. rhamnosus*, *L. casei*, *L. salivarius*); *Bifidobacteria* (*B. breve*, *B. lactis*, *B. longum*), *Bacillus* (*B. subtilis, B. cereus*), *Enterococcus* (*E. faecium)*, and the yeast *S. boulardii* are used as human probiotics, although in capsule or powder form rather than in food form. *Bacillus* and *Lactobacillus* differ in many characteristics. *Bacillus* and yeasts are not common components of the intestinal microflora. According to studies, most species and genera are apparently safe, but certain microorganisms can be problematic. For example, enterococci (*E. faecium* and *E. faecalis*) have emerged as opportunistic pathogens in hospital settings, causing nosocomial infections (endocarditis and bacteremia), as well as intra-abdominal, urinary tract, and central nervous system infections. Additionally, certain species and genera may carry factors for transferable antibiotic resistance, such as vancomycin-resistant *Enterococcus* strains, or bacilli—particularly those in the *Bacillus cereus* group, which are known to produce enterotoxins and emetic toxins [121]. Also, under certain circumstances, microbial metabolites of some fermented foods may pose safety risks. For example, histamine, tyramine, and other biogenic amines are formed by some LAB through the decarboxylation of amino acids during the fermentation of cheese, meat, vegetables, soy, and wine [122] and, in the absence of host-mediated detoxification systems, can provoke mild to severe effects (e.g., migraines) [123]. Mycotoxins are also a potential concern for all fermented foods produced with filamentous fungi. However, through domestication and careful strain selection, mycotoxin-producing lines of *Aspergillus* and *Penicillium* have been effectively eliminated from koji, cheese, and other fermented foods [124,125,126]. Citrulline and reuterine, other microbial metabolites, are precursors of the toxic compound’s ethyl carbamate [127] and acrolein, respectively [128]. Both substances are found in both alcoholic beverages and other fermented foods, but the risks they pose to human health from consuming fermented compounds have not been established [129,130].

Fermented foods (food and beverages) are a natural source of potentially probiotic strains of LAB, and their consumption has been associated with significant health benefits (reduced risk of type 2 diabetes and cardiovascular disease [131], a putatively beneficial metabolomic profile [132]). Most likely, the main source of LAB in the human gut microbiome is these foods [133] and present potential for future probiotic development. Fermented and non-fermented food sources of future probiotics may include vegetables, cereals, fruits, honey, dairy products, meat and fish products, as well as environmental sources such as soil [134].

Beyond the key aspects of gut and immune health, emerging target conditions for probiotic therapy include liver disease [135], asthma [136], metabolic diseases [137], hypercholesterolemia [79], obesity [138], mood disorders [139], cognitive function [140], and oral health [141].

Studies have shown that traditionally fermented foods (kefir, kombucha, sauerkraut, and sourdough) may contain live microorganisms, but these may not be fully characterized in terms of the bacterial strains present, the amount of microorganism present, whether or not that amount confers a health benefit, and whether the microorganism(s) are alive at the time of consumption. In ISAPP’s opinion, these foods and beverages are not in accordance with the criteria of a probiotic product because they are mainly uncharacterized and their health benefits unconfirmed [142]. ISAPP considers that if a probiotic microorganism or a mixture of organisms is additional to a fermented food/beverage, then that food/beverage is a probiotic product [142].

It can be stated that consumer interest in fermented foods has been and is largely driven by the suggested nutritional benefits, and this interest has led to an ever-increasing popularity of these foods on almost every continent. Fermented dairy products (mainly yogurt) have been associated in epidemiological studies with reductions in adiposity factors (BMI, waist circumference) [143], a reduced risk of metabolic syndrome [144], a reduced risk of obesity [145], a reduced risk of cardiovascular disease [146] and a reduced risk of colon cancer [147]. Consumption of fermented soy products, such as miso and natto, has been associated with a reduced risk of hypertension [148] and a reduced risk of cardiovascular disease [149]. Although much of this evidence is based on prospective or epidemiological studies, over 20 randomized clinical trials (RCTs) of yogurt and fermented milk products have been reported, both in patient groups and in healthy individuals [150]. Milk kefir [151], natto [152], vinegar [153], sourdough bread [154], kimchi [155] and sauerkraut [156] have also been investigated in at least one RCT. In contrast, the evidence for health promotion for another fermented food, kombucha, is largely limited to chemical analyses and animal and cell culture models [157]. In the Dutch cohort of the European Prospective Investigation into Cancer and Nutrition (EPIC-NL) study, comprising 34 men and 409 women aged 20–70 years, consumption of fermented foods was not associated with cardiovascular disease, cancer, or overall mortality [157]. The fact is that for more than a century, microbiologists have sought to identify and describe the relevant microbial strains of fermented foods and beverages. Only in the last two decades have researchers from multiple-science disciplines (systems and molecular biology, microbial ecology, bioinformatics, genetics, genomics) begun to understand how the parts of strains are assembled to build microbial (probiotic) communities that are ultimately responsible for the attributes associated with fermented foods and beverages.

#### 4.1.3. Probiotics in the Pharmaceutical Field

Recently, probiotic-based drug delivery systems have gained much attention, as they offer additional benefits, modulation of the host immune response, inhibition of cell adhesion and invasion of pathogenic organisms, and antimicrobial activity [158,159]. Probiotics can be an economical and robust way to deliver macromolecules as they exhibit various beneficial actions such as the release of antibacterial and antimicrobial agents, thus contributing to the improvement of nonspecific immunity but also to the stimulation of macrophages [160]. These processes lead to the release of enzymes, cytokines and the increase in immunoglobulins [160] and, therefore, can be used not only as effective therapeutic adjuvants, but also as drug delivery vehicles [161]. According to the latest data published, the global probiotics market is estimated to be valued at USD 991.4 million in 2025 and is projected to reach USD 1856.8 million by 2030, growing at a CAGR of 13.4% from 2025 to 2030 [162]. The most used probiotic strains in the pharmaceutical industry are *L. plantarum* (orodispersible tablet and hydrogels), *L. casei* (powder and nasal spray), *L. acidophilus* (granules, suppositories), and *S. salivarius* (nasal spray).

Given the growing interest in the clinical application of probiotics, the Food and Drug Administration (FDA) has defined a new category of “live biotherapeutic products (LBPs)” with the aim of clarifying the regulatory status and pharmaceutical expectations [162,163]. For this reason, probiotics may be regulated differently depending on their intended use (dietary supplement or drug). Although dietary supplements do not require FDA approval, probiotics such as LBPs that are intended to be used in the prevention, treatment, or cure of disease should undergo a regulatory process like that of any new therapeutic agent [162,164]. A very important thing to keep in mind is that probiotics can lose their activity in the harsh gastric conditions of the stomach or in the presence of bile salts. In addition, various physicochemical and environmental factors during the manufacture and storage of probiotics make them non-viable [165]. For these reasons, stable probiotic formulations are needed that can overcome various pharmaceutical and biological barriers, maximizing their therapeutic efficacy and clinical applicability. Up to now, various formulation approaches have been adopted to solve the instability problems of probiotics, in particular, oral formulations that are prevalent on the current market due to their advantages (low risk of infection, cost-effectiveness and high patient compliance, including ease of self-medication) [165]. Also, other routes of administration (nasal, transdermal, vaginal, and rectal administration) have been actively pursued to optimize the therapeutic efficacy of probiotics and to provide alternative platforms for probiotic administration. Formulation strategies should vary depending on the route of administration because each route has its own biological barriers and limitations [165]. In recent years, advances in formulation techniques, including micro-/nanoencapsulation, have facilitated and are facilitating the development of more elaborate formulations with controlled particle size and surface modification, improving the viability and target selectivity of probiotics. All of this has led to improved viability and target selectivity of probiotics. In the future, effective delivery systems for therapeutic probiotics are expected to develop through advances in bioengineering, formulation technology, and material science. The combination of these techniques will create new avenues for the sophisticated and precise delivery of probiotics.

#### 4.1.4. Next-Generation Probiotics

Recently, the scientific community has recognized the crucial role of certain microbial strains in promoting human health and participating in various beneficial functions for the host. These microorganisms that inhabit the intestinal ecosystem are now referred to as next-generation probiotics and are currently considered biotherapeutic products and dietary supplements or nutraceuticals [166]. For the first time, the term next-generation probiotics (NGPs) was introduced by [167]. O’Toole et al. [167] defined NGPs as live bacteria identified based on comparative analyses of microbiota, but when administered in the correct amounts, they provide a great advantage for human health [168]. Recent advances in new research methods (metabolomics, gnotobiotics, and next-generation sequencing) have allowed a more comprehensive understanding of important topics (colonization resistance, variation in individual microbiome structures, susceptibility to external microorganisms, biogeographic diversity). These methods have also allowed studying the impact of NGPs on treatment outcomes [168]. NGPs include bacterial strains with unique health effects or genetically modified strains capable of producing therapeutic compounds. NGPs such as *Lactobacillus*, *Bifidobacterium*, *Akkermansia muciniphila* and *Faecalibacterium prausnitzii* have demonstrated promising health benefits in scientific studies: *Lactobacillus* and *Bifidobacterium* are recognized for improving gut health and immune function (certain strains have been shown to be effective in conditions such as IBS and IBD), *A. muciniphila* promotes gut barrier, function and metabolic health, and *F. prausnitzii*, is known for its anti-inflammatory properties [166]. Although the scientific community has proven the effectiveness of NGPs, further investigations are needed to determine whether the use of NGPs as preventive medicine is recommended, and this will involve the use of preclinical models and clinical trials to distinguish between all possibilities.

### 4.2. Benefits of Postbiotics

#### 4.2.1. Postbiotics–Clinical Practice

A crucial role in the production and regulation of postbiotics is played by the gut microbiome. The microbiome comprises a large ecosystem of microorganisms (bacteria, viruses, and archaea) in the human gut, playing a key role in maintaining human health by influencing human physiology. It also contributes to the regulation of digestion and metabolism, which could provide numerous nutrients, regulate energy balance, and help develop the immune system against pathogens [169,170]. To trigger molecular signals in the immune system, postbiotics can act as effector molecules [171]. To maintain the balance of metabolic, immune, and neuronal networks in the body, they enhance the absorption of nutrients and release biologically active molecules as needed [172] and their potential is related to the host organism [171]. Considering the numerous reviews on postbiotics that comprehensively highlight the general physiological functions of postbiotics, we will focus, in this subchapter, on delving into the specific proven clinical applications and their details. The most common tests conducted with postbiotics are clinical trials involving mainly the digestive system, followed by the integumentary system. Postbiotics can facilitate the direct transport of nutrients to the intestines, where they exert the greatest impact by enhancing nutrient stability and bioavailability while reducing the need for complex delivery systems. Intestinal microorganisms and their hosts communicate through two types of interactions: (1) molecular interactions following the production of bioactive metabolites (SCFAs) or (2) through interactions with host immune cells via cell surface molecules [170]. Postbiotics are effective in treating irritable bowel syndrome (IBS) and associated syndromes [173] reducing lactose intolerance, increasing the absorption of essential amines [174] and maintaining a constant secretion of mucin [175]; they have the potential to replace antibiotics in the treatment of both communicable and non-communicable diseases [176,177]. Postbiotics, because they stimulate the immune system, are associated with anti-inflammatory development in the intestine and colon, as well as immunomodulatory, antihypertensive, antiproliferative, anti-obesogenic, hypocholesterolemic and antioxidant activities [178]. In scientific literature, it has been reported that enzymes such as subtilisin-type protease and glutamyl endopeptidase are effective against the degradation and eradication of extracellular polymeric substances and biofilms generated by *Serratia marcescens*, respectively, due to the enzymatic effect of postbiotics on microorganisms [179].

One of the approaches for managing diabetic complications due to the interesting interaction between the human gut microbiota and the gut–brain axis, which includes the enteric nervous system, is postbiotics [180]. Postbiotic components (especially SCFAs—acetate, propionate and butyrate), have been shown to alleviate type 2 diabetes by maintaining intestinal homeostasis and regulating glucose metabolism after administration [180]. Beteri et al. [181] reported that postbiotics rich in SCFA and exopolysaccharides from *B. breve* 091109 facilitated a significant increase in GLP-1 and PYY production. Consequently, researchers observed an increase in insulin secretion and Hemoglobin A1c (HbA1c) levels, thus underlying the exceptional potential of postbiotics in improving symptoms in prediabetic individuals [181]. Recently, in another study, [182] used both galactopolysaccharides and exopolysaccharides from *B. breve* postbiotics in a clinical trial with prediabetic volunteers and reported a significant increase in butyrate-producing bacteria. This indicates the potential of postbiotics in managing prediabetes with minimal invasive intervention. Also, in another study, Shin et al. [182] proved that the ingestion of postbiotics, especially cell-free supernatants from probiotics such as *Bacillus velezensis*, caused anti-obesogenic effects by reducing blood cholesterol, adipose tissue and body weight [182]. In addition, it was shown that postbiotics derived from LAB fermentation (strain LKDH5) can attenuate high-fat diet-induced weight gain, adipose tissue accumulation and elevated plasma triglyceride levels. All these changes are linked to favorable changes in the gut microbiota, thus supporting the hypothesis that postbiotics influence metabolic health by modulating microbial communities [183].

Studies have shown that there is a spectrum of microorganisms producing compounds that constitute postbiotics that can influence health through the gut–microbiota–CVD axis. Nondigestible carbohydrates accessible to microbiota are metabolized by intestinal microorganisms to produce SCFA, which could be involved in host interactions for essential health benefits (in the treatment of cardiovascular diseases) [184]. To mitigate cardiovascular disease (CVD) by modulating the gut microbiota and microbial metabolites, postbiotic compounds have been investigated. For example, in mice, propionate at a concentration of 200 mmol/L led to clinically important changes, such as improvement in cardiac hypertrophy, vascular dysfunction, and a decrease in ventricular arrhythmia, aortic atherosclerotic lesion area, systemic inflammation, and cardiac fibrosis. Subsequently, supplementation with magnesium acetate at 200 mmol/L in mice caused a decrease in cardiac fibrosis, a reduction in systolic and diastolic blood pressure [185].

Postbiotic supplementation has been suggested as an alternative approach to reduce the incidence of infectious diseases in children. There are studies on the use of the heat-killed *L. acidophilus* strain for the treatment of acute diarrhea. The heat-killed *L. acidophilus* CBA L74 strain was used to prevent gastrointestinal and respiratory infections [186]. Following the results obtained in these studies, we can conclude that postbiotics provide similar beneficial therapeutic effects with probiotics, avoiding the risks associated with live microorganisms, especially in high-risk groups (children under 5 years of age).

Also, postbiotics are viewed as a feasible therapeutic alternative for allergic diseases, because they can restore the balance of T helper 1 (Th1)/T helper 2 (Th2)-mediated immune responses and support the maturation of the immune system. Postbiotics may also have beneficial effects on food allergies [33]. Bunyavanich et al. [187], following a clinical trial conducted with over 200 children, stated that the presence of a rich butyrate-producing bacterial microbiota was associated with earlier resolution of cow’s milk allergy [187].

Other studies have demonstrated the antimicrobial and antiproliferative activity of postbiotics. They possess a mixture of bioactive metabolites responsible for bacterial lysis (including bacteriocins, organic acids, hydrogen peroxide and fatty acids), all with prominent antimicrobial potential [188]. However, the composition of these soluble bioactive antimicrobial agents and their efficacy vary depending on certain factors: the strain used and the fermentation conditions [189]. For example, in the study conducted by Tong et al. [190] who evaluated the inhibitory activity on *Escherichia coli* of postbiotics. Postbiotics were obtained from solid-state fermentation, which varied depending on the combination of strains, incubation temperature and water content of the fermentation [190].

Common and preventable oral diseases related to lifestyle-induced oral biofilm dysbiosis include dental caries and periodontitis. Although there are a multitude of studies related to probiotics and prebiotics used to restore microbial balance, the role of postbiotics remains less explored. However, in vitro, studies have shown that postbiotics can inhibit biofilm formation, reduce inflammation, and suppress oral pathogens such as *Streptococcus mutans* and *Porphyromonas gingivalis* [191]. Ghafouri et al. [192] reported that postbiotics derived from aerobic oral bacteria showed promising antimicrobial activity against *Staphylococcus aureus* and low cytotoxicity against human fibroblasts. All these findings support the potential of postbiotics in the management of chemotherapy-induced oral dysbiosis and oral opportunistic infections.

Other reported studies are related to the use of postbiotics for adjuvant therapy in anticancer treatment. Thus, it was observed that postbiotics produced by *L. plantarum* exhibit selective cytotoxic effects on various cancer cells under different combinations of doses and time [193]. They did not cause toxicity or hemolysis of normal cells. Postbiotics produced by *L. plantarum* were selective towards various tumorigenic cells, suggesting that they possess certain anticancer properties and therefore have great promise as functional supplements and adjuvant therapies for anticancer treatment [194].

Recent research has also highlighted the therapeutic potential of postbiotics in bone and muscle health. For example, Jeong et al. [195] reported that KL-Biome (a postbiotic formulation derived from *L. plantarum* KM-2) improved muscle mass and function in models of dexamethasone-induced muscle atrophy. In another study by Myeong et al. [196] who investigated the postbiotic MD35, derived from *L. plantarum*, in an ovariectomized (OVX) mouse model of postmenopausal osteoporosis, they observed a significant reduction in trabecular bone loss. The structure of the femoral growth plate was also preserved. All these data suggest the anti-osteoclastogenic effects and potential as a treatment for postmenopausal osteoporosis of postbiotic MD35 [196].

Finally, in Table 2 we present several clinical studies related to postbiotics and their implications for human health.

#### 4.2.2. Postbiotics in Food

A new avenue for the development of innovative products with improved functionalities and health-promoting properties is offered by postbiotics. As previously mentioned, various research has demonstrated the efficacy of postbiotics at levels comparable to their more popular counterpart, namely probiotics. In this regard, the applications of postbiotic-based products in various industries, such as food and beverages industry, have also been studied. From this point of view, this section analyzes the current and potential applications of postbiotics in healthy foods and beverages, as well as their role in promoting the development of value-added products and addressing the changing consumer demands for natural, functional and sustainable solutions. The utilization of postbiotics in the research and development of functional foods and beverages has certain technological advantages compared to probiotics, as they are more stable and safer in the industry [202]. To meet the nutritional needs of consumers with different dietary styles (vegetarians, people allergic to milk proteins, people intolerant to lactose), there are various food products with bioactive ingredients, such as dairy and non-dairy products with the addition of probiotics [194]. Regarding the postbiotics market, according to data from Future Market Insights, the global postbiotics supplement market is estimated to reach $28.3 million by 2032, with a CAGR expected to increase from 7.6% during 2016–2021 to 11.5% during 2022–2032 [194]. According to Mishra et al. [203], the use of postbiotics as natural or bio-based preservatives, postbiotic-enriched functional foods, bioactive compounds in food formulations, and dietary supplements aligns with the growing consumer preference for ecological products and the industry’s search for environmentally friendly and sustainable ingredients [204]. In this regard, this is due to growing concerns about the potential long-term effects and individual sensitivities associated with conventional food preservatives (butylhydroxyanisole-BHA and butylhydroxytoluene-BHT, nitrites, nitrates, and sulfites) [204]. For instance, Hamad et al. [205] demonstrated that the postbiotic preparation consisted of a filtered cell-free culture supernatant derived from *L. rhamnosus* EMCC 1105, at a dose of 100 mg/g, significantly reduced *Clostridium perfringens* in minced chicken meat [205]. Similarly, the cell-free supernatant of a strain of *L. plantarum* was used as a biopreservative, being effective for chicken breast filets contaminated with *E. faecium* 711, maintaining the integrity of the food product for over a week [206]. However, studies with postbiotics and food products are still scarce and focused on cereal-based products [207], beverages [208,209,210] and infant formulas [211]. In a rat study (n = 21), conducted by Almada et al. [207], administration of durum wheat pasta with gamma-irradiated *B. animalis* resulted in decreases in cholesterol and glucose levels and improved gut microbiota (higher numbers of *Actinobacteria* and *Firmicutes* and lower numbers of *Bacteroidetes*). Extending postbiotic use beyond cereal products and beverages to process meats, seafood, and other complex foods presents challenges related to matrix interactions, stability, sensory effects, and regulatory compliance. Nonetheless, laboratory and pilot-scale evidence support their potential as natural, bioactive preservatives, particularly when integrated with complementary preservation strategies or encapsulation technologies. Further research is needed to optimize formulations, validate efficacy under industrial conditions, and address regulatory pathways for commercialization [36].

The most widespread process with postbiotic applications is fermentation, and *Lactobacillus* and *Bifidobacterium* strains are commonly used as producer strains [5]. The dairy industry benefits most from the exopolysaccharides (EPS) specific to dairy starter cultures, as they reduce moisture content and have significant control over the rheological characteristics of fermented dairy products [212]. Also, increasing vitamin B levels and decreasing toxic components during probiotic-induced fermentation is another innovative approach [5].

Considering the published data, industrial postbiotic production is the systematic optimization of fermentation parameters. Empirical approaches such as Plackett–Burman design, response surface methodology (RSM) and central composite design (CCD), such methods allow producers to identify and fine-tune critical variables—such as carbon and nitrogen sources, temperature, pH, inoculum size, and trace elements—that significantly influence metabolite synthesis and total yield. In addition to media and process optimization, controlled fermentation system design is another industrial tactic. Scaling from small-volume systems to bioreactors with precise control of anaerobic conditions, mixing (agitation), gas transfer, and shear stress improves reproducibility and functional activity maintenance during production runs. These parameters influence both biomass accumulation and metabolite secretion dynamics, ensuring consistent quality and facilitating process scale-up. Finally, downstream processing and stabilization such as microencapsulation, controlled inactivation (thermal or non-thermal), freeze-drying, and advanced filtration help preserve biological activity post-fermentation and extend product shelf life. These methods also enhance functional stability in end-use applications (e.g., food, pharmaceuticals, nutraceuticals), thereby maintaining bioactivity while facilitating storage and formulation. Taken together, these industrial approaches—statistical optimization of culture conditions, controlled bioreactor fermentation, metabolic pathway engineering, and targeted downstream enhancement—constitute the most promising avenues for improving postbiotic yield and functional consistency at scale. Continued integration of multi-omics characterization and automated process control will further bolster efficiency and product quality in industrial settings.

#### 4.2.3. Postbiotics in Pharmaceutical Field

Like the food industry, the pharmaceutical sector has had and continues to have an increasing interest in using postbiotics for the development of new therapeutic agents and drug delivery systems. This is since postbiotics exhibit a diverse range of bioactivities, as discussed and presented earlier in this article. Postbiotics can be considered promising therapeutic alternatives with several advantages, and recent studies have highlighted the diverse pharmaceutical uses of postbiotics. They have demonstrated potential to modulate the immune system and address gut microbiota imbalances in both children and adults or combating metabolic disorders [213], while regular monitoring and correction of lipid profile imbalances are essential to slow the progression of cardiovascular diseases [214]. In this context, postbiotics represent a promising therapeutic strategy, as they can enhance immune regulation, restore gut microbiota homeostasis, and potentially support metabolic and cardiovascular health. Such examples of the drugs produced is the product called Lacteól Fort (Laboratoire du Lacteól du docteur Boucard, France) based on heat-stabilized *L. acidophilus*, which has been shown to be effective in the treatment of acute diarrhea and IBS [215] through randomized controlled clinical trials. Another example is the study by Giordani et al. [216] who found that *L. gasseri* biosurfactants had antibiofilm capacity against methicillin-resistant *S. aureus* (MRSA).

Postbiotics such as those from *L. rhamnosus* GG have been incorporated into supplements with the aim of providing protection against oxidative stress and diarrhea in human enterocytes induced by SARSCoV-2-derived antigens [217]. Also, there has been and continues to be an increased focus on designing efficient delivery systems for postbiotics for enhanced efficacy and stability (e.g., nanoformulations and nanocarriers). In this regard, studies have shown that nanoparticles or liposomes facilitate the controlled release of postbiotics to the desired site and protect them from degradation [218,219]. The integration of postbiotics into pharmaceutical formulations by improving their stability and controlled release profiles, as well as by improving bioavailability and therapeutic efficacy, has been possible thanks to technologies such as microencapsulation and spray drying that have facilitated the obtaining of much more effective postbiotics for human health.

#### 4.2.4. Challenges in Postbiotics

As concerns about the safety of using live strains in certain patient populations (immunocompromised subjects, infants, and vulnerable patients) have arisen, the use of non-viable postbiotics as a safer option has gained popularity [219,220,221,222]. From this perspective, postbiotics are an alternative that could significantly reduce the risk of microbial translocation and infection for consumers. Calculating the percentage of dead cells in a still-viable probiotic culture is difficult and, consequently, the origin of the variation in responses usually found in live probiotic products is represented by the change in the percentage of dead cells [220,223]. In contrast, in the case of postbiotics, it is easy to demonstrate that they are devoid of any living organisms. For this reason, postbiotic-based products could have a longer shelf life and would be extremely simple to standardize [220,223]. In conclusion, postbiotic-based products would be easier to store, their shelf life could be extended, and in terms of extreme environmental conditions, logistical management would be much easier.

In recent years, researchers have identified specific postbiotic components that interact significantly with human metabolic and physiological processes to promote healthy aging, impacting health and longevity. Despite this, postbiotics still face limitations and challenges that need to be clearly addressed. This must be considered if postbiotics are to achieve their full therapeutic applications and increase patient compliance. However, even with the progress demonstrated in research (both in preclinical and clinical investigations), some studies have blurred the line between prebiotics and postbiotics, as their impact has often not been evaluated separately [71].

Now, the use of probiotics is being questioned in terms of techniques to prevent antimicrobial resistance [224,225] and the necessity to avoid long-term pharmaceutical treatments (including their adverse effects) [215]. The idea of using non-viable probiotics as an alternative therapy is increasingly accepted due to the increasing incidence of antibiotic resistance in live probiotic applications [215]. Another research challenge is related to the translation of results, due to physiological differences in gut microbiome signatures, immune signaling pathways, and environmental covariates between animal models tested and humans [20]. This may complicate the accurate mimicking of age-related conditions, hinder the generalizability of results, and make sound clinical recommendations difficult. Furthermore, the lack of knowledge about the health-modifying mechanisms of postbiotics poses another challenge in their use for specific medical conditions. More research is needed to broaden our understanding of the interplay of postbiotics with the human body/disease management mechanisms, although most postbiotic mechanisms highlighted to date are associated with modulation of the gut–brain axis [71].

Due to the inherent characteristics of postbiotics and the variability of study design, inconclusive or even contradictory results have been observed in some of the studies conducted [20]. Thus, some of the postbiotics investigated possess immunogenic properties, such as those of Gram-negative and Gram-positive cell wall components (lipopolysaccharides and lipoteichoic acids). Although some studies have demonstrated anti-inflammatory activities of lipoteichoic acid (by reducing IL-12 production and increasing IL-10 production) [226], others have reported a lack of significant anti-inflammatory activity for this cell wall component. Importantly, this component can cause damage to the intestinal membrane [227]. Discrepancies may be due to non-standardized methods of preparation of postbiotics, or derived probiotic strains, or route of administration, or doses and formulations in different studies. All these things further complicate comparisons and identification of optimal approaches for use.

Postbiotics and medicinal plants represent an innovative and natural approach to supporting gut health and the immune system. While medicinal plants are still considered rich sources of natural compounds [228] and provide bioactive compounds (phytogenics), postbiotics are beneficial metabolites produced by good bacteria, acting synergistically to balance the microbiome and reduce inflammation.

Another formidable challenge is optimizing the yield in postbiotic production to achieve maximum productivity, as it requires a delicate balance of complex factors, such as complex metabolic pathways, strain-specific variability, and meticulous fine-tuning of fermentation conditions [5]. Considering economic viability and regulatory compliance, this approach must include selecting the right probiotic strains, optimizing growth conditions, developing metabolic pathways, and implementing efficient downstream processing procedures [229]. However, when production is scaled up from the laboratory to the industrial scale, things become much more complicated [230]. To realize the full potential of postbiotics in healthcare and industry, it is essential to successfully optimize yield, foster innovation, and ensure cost-effectiveness while maintaining product quality and regulatory standards [231]. The fact that they cannot proliferate or grow within the gut and do not exert sustained effects after discontinuation of supplementation is a unique limitation of postbiotics. For this reason, innovative delivery systems are needed to extend their efficacy and reduce frequent administration [232]. In addition, research studies can rely on the quantitative estimation of novel postbiotic biomarkers correlated with positive health effects, and their quantification can help determine whether a host microbiome bio level is insufficient and whether postbiotic supplementation would be beneficial or is indicative of a medical condition [233]. 

Even though postbiotics offer promising functional and formulation advantages, unresolved gaps regarding their precise composition limit their safe application in health. They also prevent their classification as pharmaceutical agents with consistent active components. Therefore, postbiotics are more frequently developed as dietary supplements or incorporated into functional foods [234].

Finally, the concept of a personalized microbiome holds significant potential for precision medicine, despite this its application into clinical practice for probiotic and postbiotic interventions face certain challenges:

(a) Firstly, the high interindividual variability of the gut microbiome, influenced by genetic, dietary, environmental, and lifestyle factors.
(b) Second, the lack of validated clinical biomarkers for patient stratification renders the rational process of selection of specific probiotic strains or postbiotic formulations tailored to an individual’s microbial profile difficult.
(c) Thirdly, the axis of the host–microbiome–intervention interactions, encompassing immunological, metabolic, and epigenetic mechanisms, challenge the identification of direct causal effects and necessitate integrative, multi-omics approaches.

Despite the many challenges, much of the literature addressing synergy has historically centered on synbiotics—combinations of probiotics with prebiotics designed to cooperate in the enhancement of colonization or metabolic activity—rather than on probiotic–postbiotic pairings. This reflects a broader trend in the field: despite growing acknowledgment of complementary effects across biotic categories, systematic evaluation of combined probiotic and postbiotic regimens with rigorous clinical endpoints remains limited. Therefore, critical gaps remain, including the need for:

- well-designed randomized clinical trials evaluating probiotic–postbiotic combinations against appropriate controls;
- mechanistic studies delineating whether synergy arises from enhanced host immune modulation, metabolite interactions, or microbiome-mediated pathways;
- standardized outcome measures to allow comparison across studies and conditions;

Until such data are more concretely established, the literature supports potential synergistic effects largely at the preclinical level, but insufficient clinical evidence currently exists to confirm or quantify these interactions in humans.

## 5. Limitations

Despite the comprehensive overview provided, this review has several limitations that should be acknowledged. First, the present work is a narrative, non-systematic review, which may introduce selection bias. The absence of a predefined search strategy, explicit inclusion and exclusion criteria, and a formal quality assessment of the included studies limits the ability to draw definitive conclusions regarding the strength of the evidence.

Second, a substantial proportion of the studies discussed are preclinical, including in vitro experiments and animal models. While these studies provide valuable mechanistic insights, their findings may not be directly translatable to human clinical outcomes. Moreover, many of the human studies cited are limited by small sample sizes, short intervention periods, and heterogeneity in study design, probiotic strains, dosages, and measured outcomes, which complicates cross-study comparisons.

Third, although both probiotics and postbiotics are discussed extensively, the clinical evidence supporting postbiotics remains comparatively limited. The mechanisms of action of postbiotics are not yet fully elucidated, and standardized definitions regarding their composition, effective dosage, and formulation are still lacking. This variability represents a major challenge for their clinical application and regulatory approval.

Another limitation is the predominantly optimistic focus on beneficial effects, with relatively limited discussion of negative, neutral, or conflicting results. Potential safety concerns, particularly in vulnerable populations such as immunocompromised individuals, neonates, or critically ill patients, are not extensively addressed. Additionally, issues related to long-term safety, host-specific responses, and interindividual variability remain insufficiently explored.

Finally, the rapidly evolving nature of microbiome research means that new data continues to emerge, and some conclusions presented here may require reassessment as larger, well-designed randomized controlled trials and standardized regulatory frameworks become available.

## 6. Future Perspectives

### 6.1. Probiotics

The effectiveness of probiotics can vary significantly between individuals due to genetic differences, existing gut microbiota, diet, and overall health. This variability poses a challenge in designing universally effective probiotic interventions. Future research in the field of probiotics should focus on several key areas to address existing challenges and explore new opportunities. These include:

(a) development of new strains: There is a need for continuous exploration of new probiotic strains from diverse sources, including the human microbiome, fermented foods, and natural environments. The discovery of new strains may bring unique health benefits and expand the range of interventions available for human health.
(b) brain–gut–microbiota interactions: Research should focus on understanding the complex interactions in this axis, including how these interactions influence health and disease. This will contribute to and facilitate the identification of key microbial functions and pathways that can be targeted by therapeutic interventions.
(c) studies related to the mechanism of action: Much more detailed studies are needed to elucidate the mechanisms by which probiotics exert their effects on the host. Once these mechanisms are fully understood, we will obtain complete information about their therapeutic potential, and this will lead to the development of much more effective interventions.
(d) legislation: It is essential to establish clear regulatory frameworks for the evaluation and approval of probiotics, including the establishment of standards for quality control, food safety assessment and clinical efficacy.
(e) more clinical trials: These are essential to establish the efficacy and safety of probiotics for various conditions. These studies should be much more rigorous and better designed, include diverse populations, and considering various factors such age, sex, genetics, and diet that influence human health.

### 6.2. Postbiotics

Postbiotics show promise as potential nutraceuticals for human and animal health enhancement. To combat antimicrobial drug resistance problems existing medications and the antimicrobial active postbiotic molecules can be administered with available drugs. Microencapsulated postbiotic molecules can be administered to address several metabolic syndromes, which can be analyzed by bioimaging systems. Screening tools can also be developed to authenticate intestinal products, and future techniques should determine the quantity and quality of intestinal microorganisms, as well as the mechanism of their release. But most importantly, the new methodology should validate the microbiomes of sick individuals. Another important aspect is the need for more research and clinical studies in order to increase control over the safety issues of postbiotics. It is mandatory to establish industrial standards (national or international) for postbiotics and improve their safety and functionality evaluation systems. Regarding technology and product development, it is also important to explore more plant-based raw materials and microbial strains suitable for fermentation, to optimize fermentation processes and to increase the yield of functional components in postbiotics. All these strategies will allow their wider application in products and will facilitate the development of new postbiotics with nutritional health benefits but also with unique flavors.

Due to their demonstrated health benefits, probiotics and postbiotics are being explored for integration into foods and health-related products. There are currently probiotic and postbiotic products available for human use on the markets; however, challenges for probiotic or postbiotic dietary supplements arise due to regulatory and standardization hurdles. The absence of consistent classification frameworks poses challenges in standardizing and ensuring the safety of probiotic or postbiotic-based interventions. Furthermore, individual variabilities in terms of microbiome signatures, dietary patterns, epigenetics, as well as patient compliance related to taste, cost, and convenience may pose issues. All these variabilities require a more holistic understanding and extensive research with new techniques such as pharmacogenomics, pharmacogenetics, nutrigenomics, nutrigenetics, bioinformatics, molecular biology, and genetically engineered, in a word, personalized medicine. In addition, the consumption of certain probiotics or postbiotics may conflict with certain religions or cultural beliefs. This poses a challenge for market access, product registration and development. In conclusion, all these aspects pave the way for future research directions focused on personalized use of probiotics and postbiotics, tailored to individual microbiome signatures and health conditions. Furthermore, we need to consider ethical best practices regarding interdisciplinary collaborations, long-term clinical trials and accessibility strategies, as probiotics and postbiotics represent the intersection of nutrition, therapeutics and well-being.

## 7. Materials and Methods

Search Strategy: A comprehensive literature search was performed across the following electronic databases: PubMed, Scopus, Web of Science, and Google Scholar. The search covered studies published between January 2000 and November 2025. Additional references were identified through citation tracking and manual searching of reference lists. The following keywords and Boolean operators were used in various combinations: “probiotics” OR “postbiotics” OR “biotics” OR health AND “gut microbiome” OR “intestinal barrier” OR “epithelial integrity” OR “immune modulation” OR “dysbiosis” OR “short-chain fatty acids (SCFA)” OR “intestinal permeability” OR “gastrointestinal diseases” AND “food Industry” OR “functional food” OR “fermented foods”. Study selection followed predefined inclusion and exclusion criteria. Only peer-reviewed studies examining probiotics, postbiotics, human health, biotherapeutic, gut microbiota, and their relevance to discussed diseases were included. Exclusion criteria comprised non-peer-reviewed materials, duplicate publications, studies with insufficient or incomplete data, and studies published outside the predetermined time interval.

## 8. Conclusions

Probiotics and postbiotics represent important and complementary strategies for modulating the gut microbiota and influencing host health. Probiotics have demonstrated beneficial effects through microbiota modulation, enhancement of gut barrier integrity, and immune and metabolic regulation, while postbiotics have emerged as promising alternatives that may offer advantages in terms of safety, stability, and formulation. However, current evidence remains uneven, with a strong reliance on preclinical studies and limited, heterogeneous clinical data. In particular, the clinical relevance of postbiotics is constrained by incomplete characterization of their mechanisms of action, lack of standardized definitions, and insufficient data on optimal dosing and long-term safety. Future progress in this field will depend on the implementation of well-designed randomized controlled trials, standardized production and characterization protocols, and improved regulatory clarity. Addressing interindividual variability and host–microbiota interactions will also be critical for translating microbiome-based interventions into effective clinical applications. Overall, while probiotics and postbiotics hold significant biotherapeutic potential, their integration into evidence-based healthcare strategies requires a more rigorous and standardized scientific foundation.

## Figures and Tables

**Table 1:** Biological Effects of Probiotic Strains on human health.

Probiotic Strain	Findings	References
*Lactobacillus* and *Bifidobacteria* spp.	improve epithelial barrier function by upregulating tight junction proteins, attenuate inflammation by inhibiting the nuclear factor kappa, enhancer of the light chain of activated B cells (NF-κB) pathway	[75,76,77]
*Lactobacillus* and *Bifidobacteria* spp.	cholesterol-lowering effects ↓ risk of CVD, antioxidant properties, anti-inflammatory action	[78,79,80,81,82,83,84]
*Lactobacillus* spp.	management of obesity and associated metabolic disorders	[85,86]
*Lactobacillus* and *Bifidobacteria* spp.	management of type 2 diabetes	[87,88,89,90]
*L. plantarum* NRIC1832 *L. plantarum* NRIC0380	Inhibition of allergy, induction of regulatory T cells by enhancement of IL-10 production	[92,93]
*L. plantarum YYC-3*	↓ inflammatory cytokines, ↓ VEGF-MMP2/9 signaling pathway, ↓ occurrence of colon tumors and mucosal damage	[94,95]
*L. acidophilus*	Improves colitis symptoms when used with *Veillonella ratti*, and upregulates protective cytokines, enhances IL-17 and IL-22 production, exhibits anti-inflammatory effects	[96,97]
*L. rhamnosus*	Helps recover body weight and colon length in colitis ↓ IL-18 levels, boosts IL-10, promotes regeneration of intestinal stem cells, improves disease markers, strengthens the epithelial barrier	[98,99]
*L. reuteri*	↑ hypothalamic expression of oxytocin, ↓ GABA receptor expression, modifies social and repetitive behaviors ↑ ICI efficacy, promoting ICI response and patients’ survival	[100,101]
*L. fermentum*	Inhibits harmful bacteria, protects against gut permeability from chemotherapy, ↓ inflammation through the NF-κB pathway, ↓ chronic gut inflammation by ↑ IL-6 and IL-10	[102,103]
*L. fermentum* JL-3	↓ oxidative stress indicators, ↓serum uric acid level (31.3%),	[104]
*Bifidobacterium breve ATCC15700*	↑ tight junction proteins, ↓ endotoxemia, maintained immune homeostasis, alleviated liver injury	[105]
*Bifidobacterium breve*	↑ goblet cell count, ↓ oxidative stress, reduces colitis symptoms, strengthens epithelial barrier	[106,107]
*Bifidobacterium longum*	↓ hypersensitivity visceral, ↑ mucosal repair, improved defecation habits	[108]
*Saccharomyces cerevisiae*	↑ IL-10, ↓ TNF-α, protects against colitis, and suppresses macrophages pyroptosis	[109,110]
*Enterococcus faecium*	↓ inflammation in obesity, and ↓ inflammation through the NF-κB pathway, enhances epithelial barrier, lowers pro-inflammatory cytokines	[111,112]
*Enterococcus faecium* CRL 183	↓ risk of CVD, antioxidant properties, anti-inflammatory action	[80]
*F. prausnitzii*	↓ disease scores and significantly reduces inflammation in IBD	[113]
*Lactobacillus*, *Bifidobacterium*, and *Streptococcus*,	Reduce the count of *S. mutans* and prevent tooth decay	[114,115]

↓ = decrease; ↑ = increase; IL = interleukin, NF-κB = nuclear factor kappa light-chain enhancer of activated B cells; TNF-α = tumor necrosis factor α; CVD = cardiovascular disease; GABA = Gamma-Aminobutyric Acid; VEGF = Vascular endothelial growth factor and MMP-2/9 = matrix metalloproteinase-2/9, ICI = Immune Checkpoint Inhibitor, IBD = Inflammatory Bowel Disease.

**Table 2:** Human Clinical Evidence on Postbiotic Interventions.

Disease	Postbiotic Intervention	Main Clinical Outcomes	References
Chronic diarrhea	Postbiotic Probio Eco^®^	Improved stool consistency, defecation frequency, reduced urgency and anxiety; ↑ beneficial gut bacteria; ↑ fecal butyrate	[197]
Metabolic health & inflammation	Postbiotic supplements (various)	↓ triglycerides, ↓ CRP, trend toward improved glycemic/insulin markers	[198]
Severe illness (CVA intensive care)	Postbiotic supplementation	↓ IL 1β, ↓ MDA, ↓ Hs CRP; reduced pneumonia incidence	[199]
Dental caries/oral health	Postbiotic products (adjunct oral hygiene)	Suggestive reductions in caries risk in children; heterogeneous evidence	[200]
Oral microbiota & immunity	Postbiotics (*Lactobacillus salivarius* derived)	↑ oral IgA, ↑ SCFA levels, ↑ microbial alpha diversity; ↓ harmful microbial abundance	[201]

↓ = decrease; ↑ = increase; IgA = immunoglobulin A; CRP = C-reactive protein; IL 1β = Interleukin 1 beta; SCFA = Short-chain fatty acids; MDA = Malondialdehyde; Hs CRP = high sensitive C-reactive protein.

## Data Availability

No new data were created or analyzed in this study. Data sharing is not applicable.

## Abbreviations

The following abbreviations are used in this manuscript:

ISAPP	International Scientific Association for Probiotics and Prebiotics
LAB	Lactic acid bacteria
NGPs	Next-generation probiotics
AAD	Antibiotic-associated diarrhea
GI	Gastrointestinal tract
NAD	Nicotinamide Adenine Dinucleotide
NADP	Nicotinamide Adenine Dinucleotide Phosphate
SCFAs	Short-chain fatty acids
NEC	Necrotizing enterocolitis
RWD	Real-world data
HDL-C	High-density lipoprotein cholesterol
LDL	Low-density lipoprotein cholesterol
USP	Unfermented soy products
SP	Soy product
GPL-1	Glucagon-like peptide-1
PYY	Peptide YY
LPK	*Lactiplantibacillus plantarum* K50
BMI	Body mass index
CAGR	Compound annual growth rate
RCTs	Randomized clinical trials
FDA	Food and Drug Administration
IBS	Irritable bowel syndrome
HbA1c	Hemoglobin A1c
Th1	T helper 1
Th2	T helper 2
OVX	Ovariectomized
BHA	Butylhydroxyanisole
BHT	Butylhydroxytoluene
EPS	Exopolysaccharides
MRSA	*Staphylococcus aureus* Methicillin-resistant
RSM	Response surface methodology
CCD	Central composite design
AGA	American Gastroenterological Association
NPC1L1	Niemann-Pick C1-like 1
LXR	Hepatic X receptor
